#  Evaluation of a Master of Science in Integrated Clinical and Community Mental Health (MSc ICCMH) program in Ethiopia

**DOI:** 10.3205/000266

**Published:** 2018-12-18

**Authors:** Matiwos Soboka, Kristina Adorjan, Sandra Dehning, Tsedeke Asaminew, Mubarek Abera, Matthias Siebeck, Markos Tesfaye, Andrea Jobst

**Affiliations:** 1 Department of Psychiatry, College of Health Sciences, Jimma University, Jimma, Ethiopia; 2 Center for International Health, LMU, Munich, Germany; 3 Department of Psychiatry and Psychotherapy, LMU, Munich, Germany; 4 Institute of Psychiatric Phenomics and Genomics (IPPG), University Hospital, LMU, Munich, Germany; 5 Department of Child and Adolescent Psychiatry, Psychosomatics, and Psychotherapy, LMU, Munich, Germany; 6 Department of Ophthalmology, College of Health Sciences, Jimma University, Jimma, Ethiopia; 7 Department of General, Visceral, Vascular and Transplantation Surgery, LMU, Munich, Germany; 8 Department of Psychiatry, St. Paul’s Hospital Millennium Medical College, Addis Ababa, Ethiopia

**Keywords:** mental health, evaluation, competency, satisfaction, non-physician clinician, brain drain, human resources, Ethiopia

## Abstract

**Introduction:** The shortage of trained manpower in the field of mental health remains a significant obstacle to the treatment of people with mental illnesses in low and middle-income countries. In 2010, a new *Master of Science in Integrated Clinical and Community Mental Health (MSc ICCMH) *program for non-physician clinicians was established at Jimma University to address this shortage in Ethiopia. This study aimed to assess the competency, satisfaction, and involvement level of graduates of the program.

**Methods:** A cross-sectional study was conducted among the graduates of the program. Data were collected with a semi-structured, self-administered questionnaire that was developed for the study. Responses were recorded on a Likert scale to assess graduates’ competency and satisfaction level. The quantitative data were summarized by descriptive statistics, including means, standard deviations, and frequencies. Qualitative data were transcribed and analyzed thematically.

**Result:** Until June 2015, 32 trainees had graduated from the MSc ICCMH program; 87.5% (n=28) of these graduates participated in the study. Almost all (96.4%, n=27) graduates were working in public institutions. The majority (75%, n=21) were directly engaged in the clinical care of patients. Also, two-thirds of the graduates (67.9%, n=19) were involved in mental health research. All of the graduates felt confident in conducting psychiatric assessments of adults and identifying and managing common mental disorders (100%, n=28). Similarly, 100% (n=28) of the graduates reported that they felt confident in identifying and managing severe mental illnesses.

**Conclusions:** The outcome of the program is a considerable workforce of skilled mental health professionals. The majority of graduates were retained within the public mental health service. Brain drain does not appear to be a challenge among non-physician mental health specialists. The findings on the self-perceived competencies mirror the amount of clinical exposure during the training. With a minimal revision of the curriculum, the level of satisfaction and competencies can be enhanced.

## Zusammenfassung

**Einführung:** Der Mangel an ausgebildeten Arbeitskräften im Bereich der psychischen Gesundheit bleibt ein wesentliches Hindernis für die Behandlung von Menschen mit psychischen Erkrankungen in Ländern mit niedrigem und mittlerem Einkommen. Im Jahr 2010 wurde an der Jimma University das neue Programm *Master of Science in Integrated Clinical and Community Mental Health (MSc ICCMH)* für nicht-ärztliches Personal ins Leben gerufen, um diesem Mangel in Äthiopien entgegenzuwirken. In der vorliegenden Studie wurden die Kompetenz, die Zufriedenheit und das Engagement der Absolventen bewertet.

**Methoden:** Unter den Absolventen des Programms wurde eine Querschnittsstudie durchgeführt. Die Daten wurden mit einem teilstrukturierten, selbstverwalteten Fragebogen erhoben, der für die Studie entwickelt wurde. Die Antworten wurden auf einer Likert-Skala erfasst, um die Kompetenz und die Zufriedenheit der Absolventen zu bewerten. Die quantitativen Daten wurden durch deskriptive Statistiken zusammengefasst, einschließlich Mittelwerte, Standardabweichungen und Häufigkeiten. Qualitative Daten wurden transkribiert und thematisch analysiert.

**Ergebnis:** Bis Juni 2015 hatten 32 Studenten das MSc-ICCMH-Programm absolviert und 87,5% (n=28) dieser Absolventen nahmen an der Studie teil. Fast alle (96,4%, n=27) Absolventen arbeiteten in öffentlichen Einrichtungen. Die Mehrheit (75%, n=21) war in der klinischen Versorgung von Patienten tätig; zwei Drittel der Absolventen (67,9%, n=19) waren auch in psychiatrische Forschungsprojekte involviert. Alle Absolventen waren zuversichtlich, psychiatrische Untersuchungen von Erwachsenen durchführen, psychische Störungen identifizieren und behandeln zu können (100%, n=28). Ähnlich berichteten 100% (n=28) der Absolventen, dass sie sich zuversichtlich fühlten, schwere psychische Erkrankungen erkennen und behandeln zu können.

**Schlussfolgerungen:** Im Rahmen des Programms konnte eine beträchtliche Anzahl von qualifizierten Mitarbeitern im Bereich der psychischen Gesundheit ausgebildet werden. Die Mehrheit der Absolventen waren im öffentlichen Dienst für psychische Gesundheit beschäftigt. Das Problem der Fachkräfte-Abwanderung scheint sich im Bereich nicht-ärztliches Fachpersonal für psychische Gesundheit nicht zu stellen. Die Studienergebnisse zu den selbst wahrgenommenen Kompetenzen spiegeln die Schwerpunktsetzung auf die klinische Expertise während des Trainings wider. Mit einer minimalen Überarbeitung des Lehrplans können die Zufriedenheit und die Kompetenzen gesteigert werden.

## Introduction

Mental disorders are on the global public health agenda because about 14% of the global burden of disease has been attributed to neuropsychiatric disorders [1]. The World Health Organization (WHO) stated that more than one third to more than one fourth of the respondents in 11 of 17 countries studied had a mental disorder in their lifetime [2]. Moreover, studies have estimated that more than 76% to 85% of adults with mental illness living in low- and middle-income countries do not receive treatment for their illness [3], [4], [5], [6], [7], [8], [9], [10]. 

The WHO has planned to reduce the burden associated with mental illness and increase the promotion of mental health worldwide [3]. In order to achieve these targets, it is mandatory to strengthen the delivery of mental health care and integrate the service into primary care [1]. However, in most countries a shortage of trained manpower in the field of mental health remains a significant obstacle with regard to the treatment of people with mental illnesses [11]. Also, very few mental health services exist in developing countries [10].

For example, in low-income countries the number of psychiatrists per 100,000 population is 0.05, compared to 8.59 in high-income countries; and the number of psychiatric nurses per 100,000 population is 0.42, compared to 29.15, respectively [11]. Similarly, in 2011, Ethiopia had only 50 psychiatrists to serve a population of over 90 million [12]. Therefore, it is crucial to train additional health professionals in the area of mental health to meet the existing need [11]. In addition, the scarcity of mental health professionals in most low-income countries, such as Ethiopia, is likely to worsen unless effective strategies are implemented [13]. Also, a workforce of skilled mental health professionals is fundamental in addressing the needs of individuals with mental illness and other psychosocial problems [13]. 

### The program

In 2010, Jimma University launched a Master in Integrated Clinical and Community Mental Health (MSc ICCMH) program in collaboration with Ludwig-Maximilians-Universität (LMU), Munich, Germany [14]. The Ethiopian Ministry of Health and other stakeholders participated in the consultative meeting for this program [14]. The program is open to candidates who hold a bachelor’s degree either in nursing or public health (health officer). Furthermore, the candidates are expected to have at least two years of work experience before they join the program. Students attend a full academic schedule with didactic lectures, problem-based learning tutorials, and interactive case sessions. The program aims to generate non-physician mental health specialists who can perform the various clinical, academic, administrative, and research activities within the field of mental health. However, the program has not yet been evaluated as to whether the graduates’ competencies meet the mental health needs of the population. Also, no information is available regarding graduates’ level of satisfaction with the program in relation to their working situations. It is crucial to evaluate the program (i.e. its curriculum) and the clinical competency of graduates to allow for the curriculum to be revised if need be. Moreover, obtaining feedback will help support graduates’ professional development and reduce future challenges in the field, and will result in more competent professionals, in line with the goal of scaling up mental health resources [13], [14], [15], [16], [17].

### Courses

The following courses are delivered as lectures in blocks:

- applied neuroscience;
- general adult psychiatry;
- social work and family assessment;
- normal psychology and psychological development;
- psychopharmacology;
- research methods in mental health; child and adolescent psychiatry;
- social determinants of health;
- ethics, law and professionalism in psychiatry;
- counseling psychology;
- special topics in psychiatry;
- principles of psychotherapy;
- mental health services management;
- clinical addiction psychiatry.

The clinical parts of the program are as follows:

- clinical psychiatry I, II, III, and IV;
- child and adolescent psychiatry;
- consultation-liaison psychiatry.

Clinical psychiatric training takes place at the Psychiatric Clinic of Jimma University Teaching Hospital. The above mentioned modules form the core curriculum and feature as part of the Master of Science in Integrated Clinical and Community Mental Health (MSc ICCMH) curriculum in Ethiopia. The program takes two years and has a total of 61 credit hours. Students do not have to pay tuition fees, but they are obliged to serve in public institutions after completing the program.

Curriculum models are very important to provide a conceptual framework for designing a particular evaluation, depending on its specific purpose. We reviewed four such models to see if they were suitable for evaluating the MSc ICCMH program:

1. Tyler’s Model, which measures students’ progress towards instructional objectives. This model specifies instructional objectives, collects performance data and compares performance data with the objectives/standards specified. However, this model ignores the process and fails to diagnose reasons why a curriculum has failed [18].
2. CIPP Model, which concentrates on the *context* of the program, the *input* into the program, the *process* within the program, and the *product* of the program. This model focuses on decision-making for goals, procedural designs, procedures in use, and attainments. However, this model overvalues efficiency and undervalues students’ aims [19], [20].
3. Stake’s Model, which emphasizes the description and judgement of data. This model stirs up value conflicts and ignores causes [18].
4. Kirkpatrick Model, which helps trainers measure the effectiveness of their training in an objective way [18].

### Research question

This study aimed to assess the MSc ICCMH program with respect to graduates’ competency, satisfaction with the program and current work position, and involvement in public mental health services, and to obtain suggestions from graduates on how to further improve the program.

## Methods

### Study setting 

The study was conducted among MSc graduates of the MSc ICCMH program from Jimma University in Ethiopia. Jimma University is one of the leading universities in Ethiopia and located in the Southwest part of the country. Jimma University and Ludwig-Maximilians-Universität (LMU), Munich, Germany, jointly launched this 2-year MSc program in mental health in 2010. Applicants from all over Ethiopia have enrolled in the program. All program participants who had graduated by the end of 2015 were invited to participate in the study.

### Study design

This was a cross-sectional study. No sample size calculation or sampling procedures were necessary because we aimed to include all graduates from the program. 

### Assessments

Data were collected from graduates in November 2015 by a self-administered questionnaire. Data collection was supervised by a psychiatrist. 

### Socio-demographic variables

We assessed the following socio-demographic variables: age, sex, marital status, region, employment status, years and months of practicing psychiatry after graduation, previous background (background training in psychiatry, e.g. as a psychiatric nurse, general nurse, health officer, midwife), previous experience in mental health, work place, and work position.

### Outcome variables

Outcome variables were assessed with a questionnaire developed on the basis of the curriculum for the graduate program. To assess the subjective level of confidence in competency areas, graduates gave Likert-type responses as follows: 1 (very low), 2 (low), 3 (fair), 4 (good), and 5 (excellent). Participants who self-rated a score =3 were considered to be less competent in the respective area of competency. Likert-type responses were also used to assess graduates’ satisfaction with the program and with their current working position as follows: 1 (very dissatisfied), 2 (dissatisfied), 3 (neutral), 4 (satisfied), and 5 (very satisfied). Participants who scored =3 were considered to be unsatisfied with the respective feature of the program. Finally, we used an open-ended question to assess graduates’ current activities.

### Data processing and analysis

Quantitative data were analyzed by descriptive statistics, including frequencies, means, and standard deviations. Graduates who scored =4 on competency questions were considered to be competent in the respective area. Similarly, graduates who scored =4 on satisfaction questions were considered to be satisfied in that area. The responses to the open-ended questions were grouped according to the evaluation questions and topics for thematic evaluation. Data were read line by line in order to describe and cluster them, and to find commonalities and examine different ideas for analyzing them thematically. 

### Ethical considerations

The study was approved by the Ethical Review Board of the Jimma University College of Health Sciences. Written informed consent was obtained from study participants. Participants’ privacy was ensured during data collection, and participants’ data were kept anonymous at every stage of data analysis and interpretation. Participants had the right not to participate in the study and to withdraw from the study at any time. 

## Results

### Response rate

By June 2015, 32 students had graduated from the program at Jimma University; all of the graduates were invited to participate in the study. Two of the graduates were general practitioners from Somaliland; after graduating from the MSc ICCMH program, they returned to their country to work as mental health specialists and unfortunately were unable to participate in the study. The majority of the graduates (87.5%, n=28) participated in the study. 

### Graduates’ characteristics 

**Figure 1:**
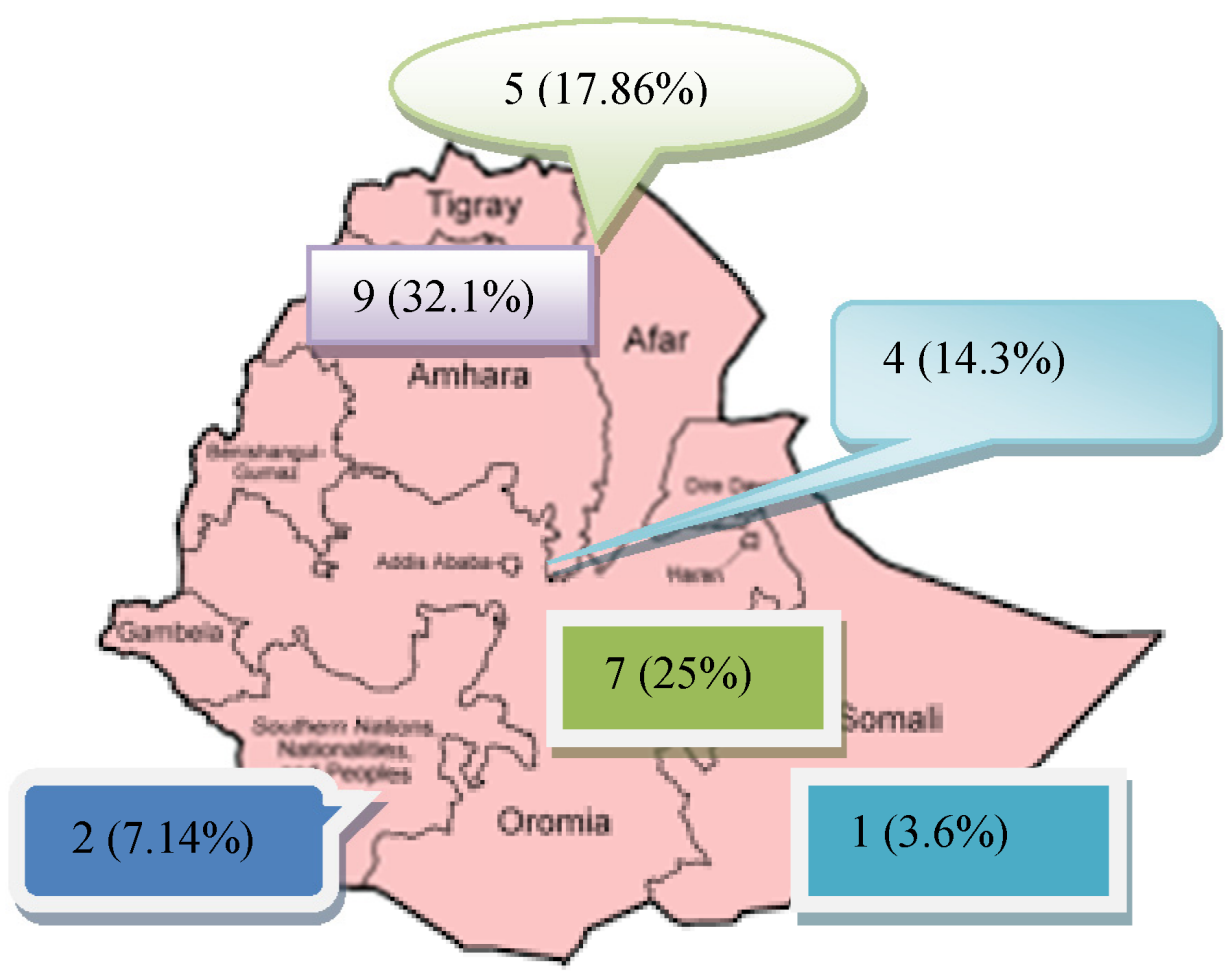
Regions and cities in which graduates are currently working, 2015

Table 1 (Tab. 1) shows the graduates’ socio-demographic characteristics. The majority of study participants (82.1%, n=23) were male and just over half were married (53.6%, n=15). The mean age of the participants was 31.5 years (SD 6.7, range 26–50 years). Before joining the program, the majority of graduates (60.7%, n=17) had obtained a bachelor’s degree in nursing, followed by health officers (35.7%, n=10). At the time of the study, almost two thirds of the graduates (64.3%, n=18) were working in universities, followed by health care facilities (17.9%, n=5); nearly all graduates were working in public institutions (96.4%, n=27), and only one graduate was working in a non-governmental institution (3.6%, n=1). At the time when the study was conducted, graduates’ mean number of service years since having graduated from the program was 1.9 (SD 1.3), and the maximum number of service years was 4. The graduates were working in different parts of Ethiopia (see Figure 1 (Fig. 1)).

### Graduates’ level of confidence in competency areas

All of the graduates (100%, n=28) were confident in their ability to conduct a psychiatric assessment of adults and identify and manage common mental disorders. Similarly, all of them (100%, n=28) reported that they felt confident in identifying and managing severe mental illnesses. The majority reported that they felt confident in conducting comprehensive psychiatric assessments of children and adolescents (82.1%, n=23) and in identifying and managing substance use disorders (89.3%, n=25). A high percentage of the graduates reported that they felt confident in educating and counseling caregivers of patients with mental illness (89.3%, n=25) and in recognizing available resources in their area and referring patients to specialized services when needed (92.9%, n=26). Also, 92.9% (n=26) of the graduates felt confident in improving the capacity of their institution to provide mental services. Graduates felt confident in practicing leadership skills (82.1%, n=23) and management skills (96.4%, n=27), undertaking research to assess the mental health needs of a given community (82.1%, n=23), and effectively communicating with the community (89.3%, n=25). Moreover, 82.1% (n=23) of graduates reported that they felt confident in identifying and managing common medical problems (see Table 2 (Tab. 2)).

### Perceived strengths and weaknesses of the program 

Allocating sufficient time for clinical practice (46.4%, n=13), the presence of effective and committed teachers (17.9%, n=5), providing community mental services (14.3%, n=4), collaboration with LMU (7.1%, n=2), the inclusion of a night-duty program (7.1%, n=2), strong supervision (3.6%, n=1), participation in morning sessions (3.6%, n=1), bed-side teaching (3.6%, n=1), ward rounds (3.6%, n=1), comfortable teaching environment (3.6%, n=1), and availability of beds and psychotropic medications (3.6%, n=1) were some of the strengths mentioned by graduates. The weaknesses listed by graduates included less priority given to research (25%, n=7), lack of incentives for the night-duty program (14.3%, n=4), too many contact hours in the program (61 credit hours; 7.1%, n=2), unclear schedule (3.6%, n=1), lack of coordination (3.6%, n=1), and few students per year (3.6%, n=1).

### Graduates’ perception of the taught courses 

Graduates perceived the following courses as being the least beneficial: social determinants of health (28.6%, n=8), health service management (10.7%, n=3), counseling psychology (7.1%, n=2), social work (10.7%, n=3), and developmental psychology (3.6%, n=1). They considered courses such as adult psychiatry (39.3%, n=11), child and adolescent psychiatry (17.9%, n=5), addiction psychiatry (7.1%, n=2), clinical attachment (39.3%, n=11), psychopharmacology (17.9%, n=5), and applied neuroscience (7.1%, n=2) to be the most beneficial. Suggestions for courses to be included in the program were neurological disorders, biostatistics, and epidemiology. Graduates suggested that child and adolescent psychiatry should have more credit hours and a clinical attachment for psychotherapy should be added. “Neurological disorders, especially clinical attachment in neurological disorders, should be included in the program because most patients with neurological disorders are seen at a psychiatry clinic” (ID #1). Similarly, another participant reported that “In my opinion, adding a clinical attachment in psychotherapy is very important because we didn’t get a chance to learn how to provide psychotherapy to patients” (ID #4).

Some graduates liked the following teaching methods: clinical attachment (28.6%, n=8), practical teaching (14.3%, n=4), video teaching of child and adolescent psychiatry (10.7%, n=3), morning sessions (25%, n=7), ward rounds (21.4%, n=6), seminar presentation (10.7%, n=3), case-based teaching (14.3%, n=4), method of teaching addiction psychiatry (3.6%, n=1), and bed-side discussions (3.6% n=1). However, some did not like the tight schedules of clinical practice (7.14%, n=2), long ward rounds (7.14%, n=2), lectures without practical sessions (7.14%, n=2), and long morning sessions (3.6%, n=1).

### Graduates’ perception of student assessment methods used in the program

Graduates reported that they liked some of the assessment methods, including the written exams (32%, n=9), case-based clinical examinations (32%, n=9), and progressive assessment (10.7%, n=3). However, some of them (17.9%, n=5) disliked the progressive assessments and case-based clinical examinations. Similarly, 7.1% (n=2) and 10.7% (n=3) of the graduates disliked observed clinical examinations and written exams, respectively (see Table 3 (Tab. 3)). One graduate reported that “[i]nterviewing a patient in front of examiners during the clinical examination is too subjective and makes examiners biased” (ID #9).

### Suggestions for improving the program

Important suggestions for improving the program included the following: payment as an incentive for night duty (25%, n=7); reducing the clinical practice time (14.3%, n=4); practice-based research methodology (14.3%, n=4); adding clinical practice in addiction psychiatry (10.7%, n=3); increasing credit hours for neuroscience (10.7%, n=3); increasing credit hours for psychopharmacology (7%, n=2); listening to students’ complaints and giving students more free time (3.6%, n=1); including Mental Health Gap Action Program (mhGAP) training [9] in the program (3.6%, n=1); offering conditions that enable students to complete the program within 2 years, as intended (3.6%, n=1); increasing the duration of the child and adolescent psychiatry clinical attachment (3.6%, n=1); emphasizing clinical practice in neurological disorders (3.6%, n=1); and skills training in electroencephalogram (EEG), electroconvulsive therapy (ECT), and psychotherapy (3.6%, n=1).

### Graduates’ activities at the time of the study

#### Clinical services

Three fourths (75%, n=21) of the graduates have been involved in providing clinical psychiatric services as follows: conducting psychiatric assessments of children and adolescents (39.3%, n=11) or adults (67.9%, n=19); identifying and managing substance use disorders and medical problems among psychiatric patients (60.7%, n=17), severe mental illness (71.4%, n=20), and common mental disorders (75%, n=21); educating and counseling patients and their caregivers (71.4%, n=20); and effectively consulting senior colleagues (64.3%, n=18), other health professionals (71.4%, n=20), and colleagues (75%, n=21) for difficult cases.

#### Research activities

Over two thirds of the graduates (67.9%, n=19) were involved in research activities. The reasons given by the remaining graduates for why they were not involved in research activities included lack of motivation, short period since graduation, difficult communication with potential mentors, lack of funds at the hospital where they were working, and lack of interest. Among all graduates, 39.3% (n=11) had between 1 and 13 scientific publications, including their thesis. However, 60.7% (n=17) of them did not have a single publication, including their thesis; one of these graduates reported that “[t]he reason for not having published is personal limitations on allocating time” (ID #25).

#### Teaching

The majority (78.6%, n=22) of graduates were involved in teaching activities in a university or college, and a significant proportion (17.9%, n=5) were supervising research projects by various students. Many graduates (39.3%, n=11) were working in a leadership position, 27.3% (n=3) and 72.7% (n=8) of them as heads of departments or coordinators of various programs, respectively. Moreover, 14.3% (n=4) and 21.4% (n=6) of the graduates were engaged in providing mental health training and community services, respectively. The majority of the graduates had stayed within public institutions and were engaged in training other health professionals and providing mental health care.

### Graduates’ level of satisfaction with the program and their current working position

Almost all of the graduates (96.4%, n=27) reported that they felt satisfied with the program’s teaching methods and courses, and 85.7% (n=24) were satisfied in general with the program in its current form. Most of them (92.9%, n=26) were satisfied with the clinical attachment, and 71.4% (n=20) were satisfied with liaison psychiatry. However, more than one third of graduates (35.7%, n=10) did not feel satisfied with the program’s assessment methods. Similarly, one fourth (25%, n=7) were not satisfied with their current working position, discontinuity due to the lack of a PhD program (3.6%, n=1), and unclear job descriptions after graduation (3.6%, n=1).

## Discussion

Since the response rate was high (28 of 32 students participated), the data can be considered to be representative.

Our findings indicate that non-physician graduates of the MSc ICCMH program at Jimma University, Ethiopia, gain confidence in general adult psychiatry, including the assessment and management of severe mental illnesses and common mental disorders, counseling patients and their caregivers, and effectively consulting other health professionals. Sufficient clinical practice throughout the semester and graduates’ satisfaction with the clinical activities may be important reasons why graduates were confident in managing common and severe mental disorders. Most of the graduates were satisfied with the program’s teaching methods.

The majority of the graduates felt that they have good management skills and can thus improve the mental health services of the community in which they are working. Sufficient involvement and exposure of graduates to management issues of the department during their training may be a reason why graduates have good management skills. This is a relevant competency because more than one third of graduates are currently working as the head and coordinator of various programs.

The majority of graduates also felt that they have good communication skills to interact with the community. The graduates’ participation in the developmental team training program (DTTP) course might have contributed to enhancing their skills in communicating with the community. During DTTP, students from different disciplines come together, stay in the community for two months, and try to identify and solve health and health-related problems by working together with community leaders [21]. Through working in the community, students get the chance to learn how to assess and prioritize community problems and plan interventions for them via effective communication with the community and students from different disciplines. Also, program participants have the chance to acquire skills to work in teams with different health professionals.

In this evaluation, we found that the graduates felt confident in their communication skills with other staff; this might have been acquired while they were working with nurses, runners, and hospital porters during their clinical attachment.

Even though graduates were confident in clinical psychiatry, nearly one fifth (17.9%) reported that they were not confident enough in conducting psychiatric assessments of children and adolescents. This low level of confidence may be due to the low exposure to children and adolescents with mental illness during the program. Since specialist child and adolescent psychiatry services are very few, and limited to the capital city of the country [22], graduates only had one month of clinical attachment for child and adolescent psychiatry [14]. After graduating, they did not have a chance to practice regularly what they had learnt theoretically. 10.7% of graduates reported they were not confident enough in identifying and managing substance use disorders. Much like specialist child and adolescent psychiatry services, specialist centers for the treatment of substance use disorders are also limited to the capital city of the country. Therefore, increasing the time allocated in the program to clinical practice for child and adolescent psychiatry and addiction psychiatry may enhance trainees’ level of confidence in these fields.

In Ethiopia, mental health services are inadequate, and there is a scarcity of human resources for mental health. The lack of access to services, the lack of awareness with regard to mental health issues, and the stigma associated with mental illness contribute to the suffering of many patients who are kept at home and even chained up [17]. The lack of mental health professionals represents a further obstacle to the expansion of mental health services [17], [22]. Ethiopia has a population of more than 90 million, but currently only 50 psychiatrists practice psychiatry in the country. Also, there are no more than 461 psychiatric nurses [17]. Therefore, to increase the number of skilled professionals in the area of mental health, in 2010 Jimma University introduced the MSc ICCMH program, developed in collaboration with LMU, to produce highly competitive, non-physician mental health professional specialists [23], [24]. The two universities are working together to improve mental health services in Ethiopia. LMU sends instructors to Jimma University, teaching courses on addiction, child and adolescent psychiatry, neuroscience, psychopharmacology, mental health ethics and law, and special topics in psychiatry. The remaining courses are taught by instructors from Jimma University and other collaborating institutions. 

Since 2010, 32 students have graduated from the program and are working in different health institutions, universities, and non-governmental organizations. Graduates are expected to perform many clinical activities, to supervise mental health services, and to scale up mental health services in Ethiopia [17], [25].

In this study, graduates acknowledged the following strengths of the program: sufficient clinical practice, a night-duty program, provision of community services, collaboration of Jimma University with LMU, and the presence of committed instructors. Graduates stated the teaching style of the program, including the clinical attachment, as a further positive factor. The program participants may have liked this aspect because the program is student-centered and the students fully participate in the teaching/learning process. Graduates’ suggestions and comments are the major source of ideas for further improving the program.

Yet there are also some contradictory, more critical commentaries: too many courses; no time; course should be finished on time after 2 years; a series of courses should be added. A solution could be continued professional development courses focused on specific areas.

### Current work situation

Since graduating, three quarters of the program participants have been providing clinical mental health services in different hospitals. Most of the graduates have been playing a key role in the expansion of mental health services in the country by providing mental health training (mhGAP) [9] and supervision for primary health care workers.

The majority of the graduates are working in Ethiopia, and brain drain will not be a problem among these non-physician mental health specialists, because most of them were trained in their home institution. However, graduates are dissatisfied with the lack of job descriptions and of a PhD program.

Generally, the findings of the study imply that in developing countries comprehensive mental health services can be delivered by non-physician mental health specialists. Also, they imply that there is a need to revise the MSc ICCMH program to enhance the quality of the training.

This study is limited by the fact that we used our own, unvalidated questionnaire to evaluate the program because we could not find a suitable validated tool. Our assessment of graduates’ competency was based on a self-assessment, which may be biased and therefore not reflect competence levels accurately.

Overall, the outcome of the program is a considerable workforce of skilled mental health professionals.

## Conclusions

The majority of graduates of the MSc ICCMH program rated themselves as being clinically competent, which is highly valuable in improving mental health services in Ethiopia. The findings on the self-perceived competencies mirror the degree of clinical exposure during training. There is a need for more clinical training in child and adolescent psychiatry. The majority of graduates stayed within the public mental health service, and brain drain does not appear to be a challenge among these non-physician mental health specialists. The findings can greatly contribute to improving the quality of mental health services through revision of the curriculum for training non-physician clinicians. 

## Notes

### Acknowledgements

We are grateful to the Center for International Health, LMU Munich, for their support of this program. 

We thank Jacquie Klesing, Board-certified Editor in the Life Sciences (ELS), for her editing assistance with the manuscript. Also, we extend gratitude to the study participants. 

### Funding

This study was funded by the Center for International Health, LMU Munich.

### Availability of data and materials

Data is available upon request to the corresponding author.

### Authors’ contributions

Matiwos Soboka and Kristina Adorjan contributed equally to the manuscript and have shared first authorship. Markos Tesfaye and Andrea Jobst have shared last authorship.

Matiwos Soboka contributed to the design, conduct and analyses of the research and to the manuscript preparation. Kristina Adorjan contributed to the design, conduct and analyses of the research and to the manuscript preparation. Sandra Dehning, Tsedeke Asaminew, Mubarek Abera, Matthias Siebeck, Markos Tesfaye, and Andrea Jobst contributed to the design, conduct and analyses of the research and to the review of the manuscript. All authors read and approved the manuscript.

### Competing interests

The authors declare that they have no competing interests.

### Ethics approval and consent to participate

Ethics approval was obtained from the Institutional Review Board of Jimma University Health Sciences College. Written informed consent was obtained from all graduates who agreed to participate in the study.

## Figures and Tables

**Table 1:**
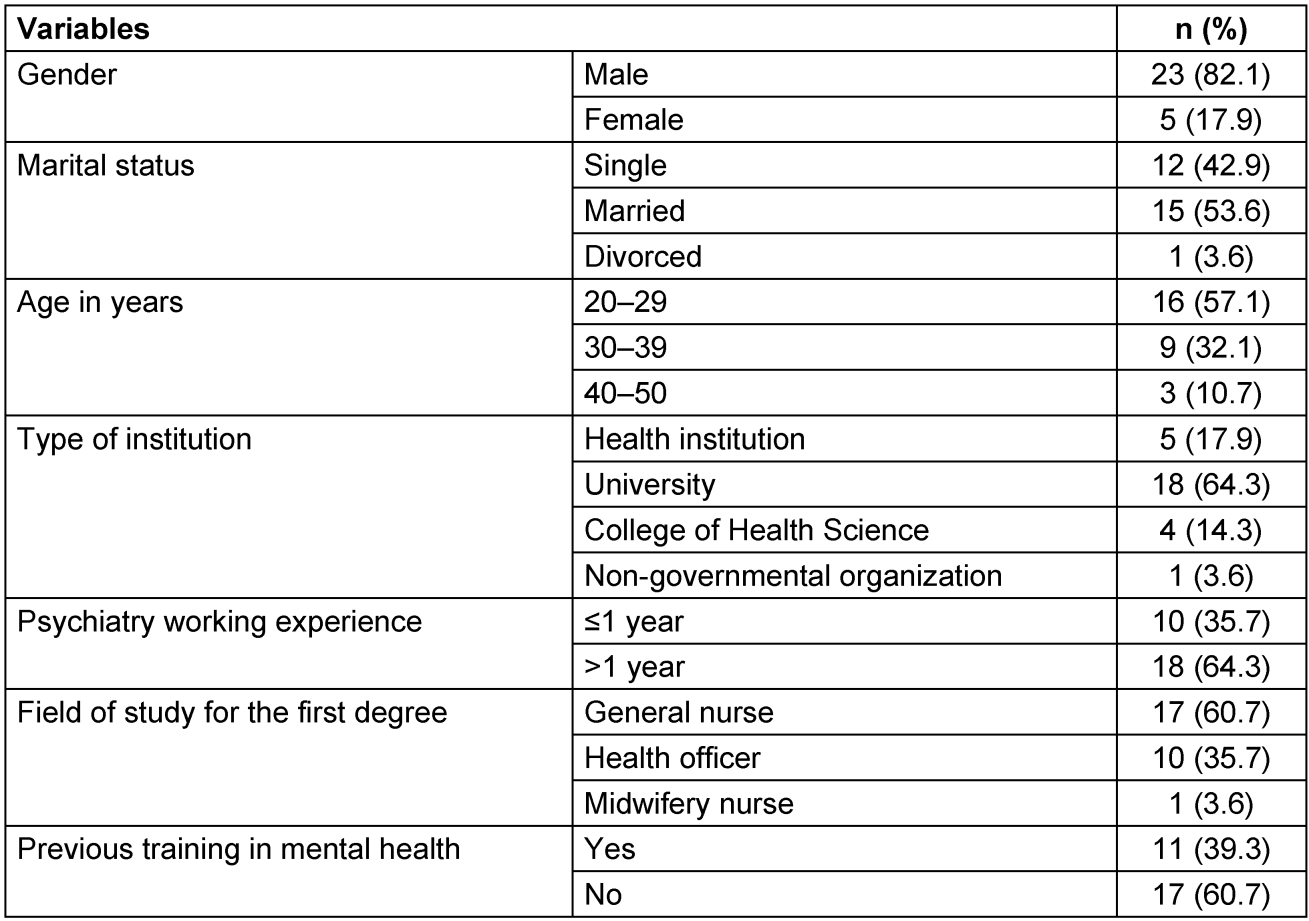
Socio-demographic characteristics of graduates from the Master of Science in Integrated Clinical and Community Health (MSc ICCMH) program, 2015

**Table 2:**
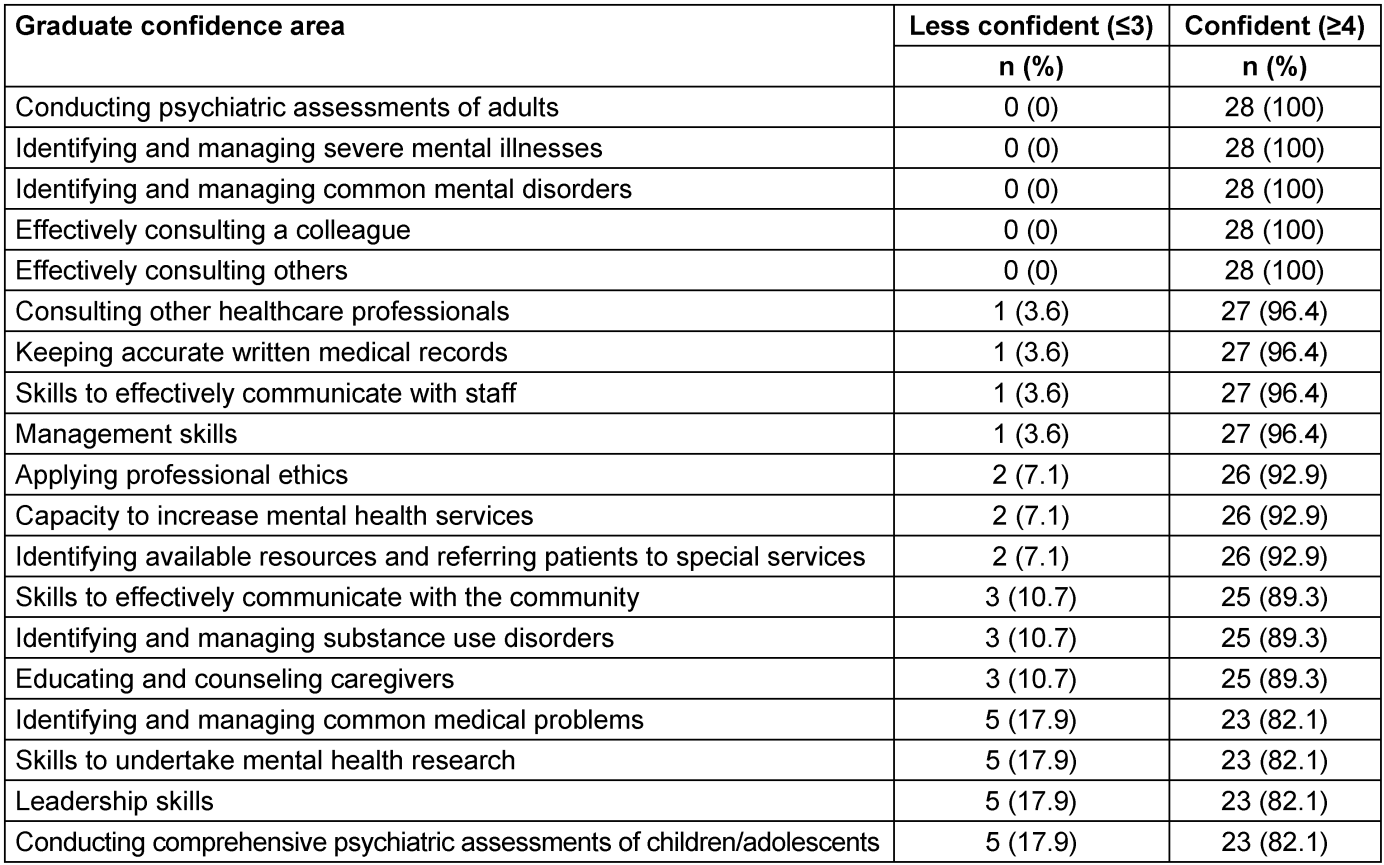
Competencies of graduates from the Master of Science in Integrated Clinical and Community Mental Health (MSc ICCMH) program, 2015

**Table 3:**
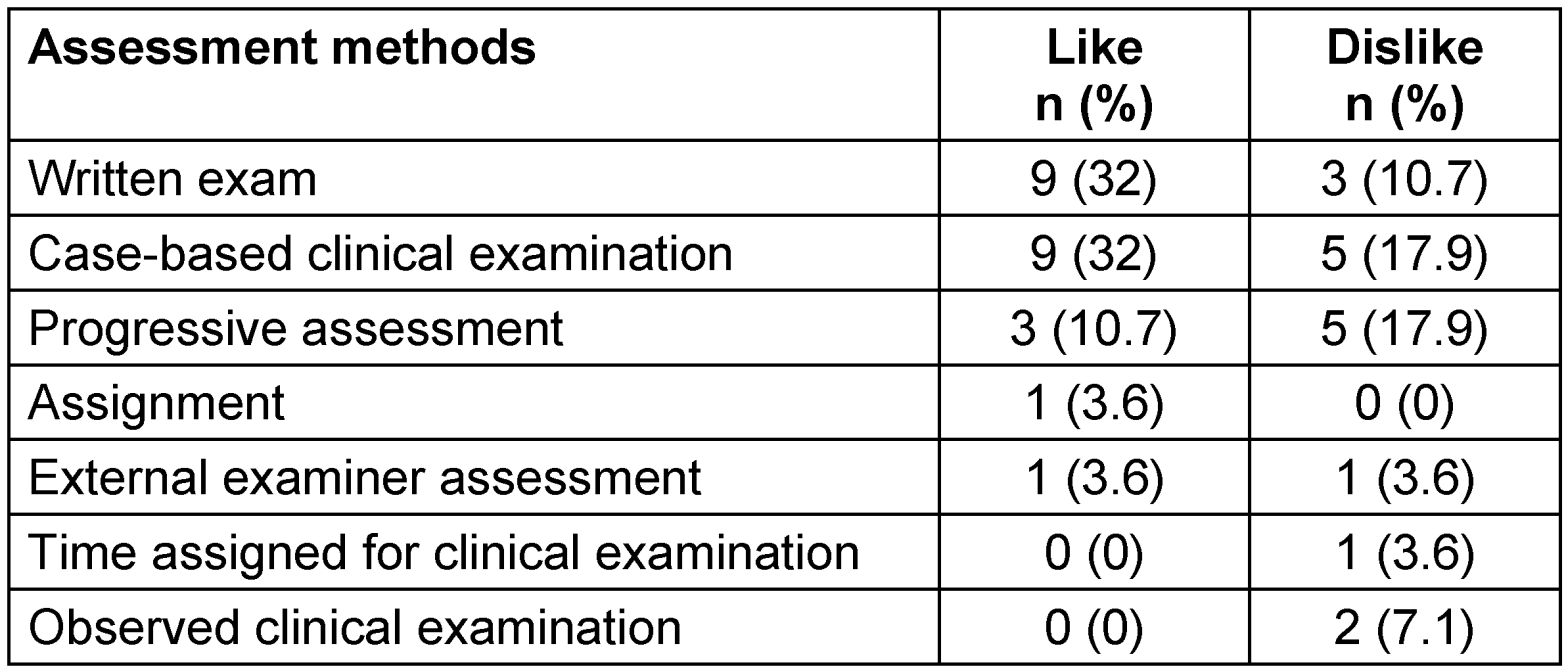
Acceptability of the assessment methods by graduates of the Master of Science in Integrated Clinical and Community Mental Health (MSc ICCMH) program, 2015
